# The rise of checkbox AI ethics: a review

**DOI:** 10.1007/s43681-024-00563-x

**Published:** 2024-09-05

**Authors:** Sara Kijewski, Elettra Ronchi, Effy Vayena

**Affiliations:** 1 Department of Health Sciences and Technology, Chair of Bioethics, ETH Zurich, Zurich, Switzerland; 2 Data and Digital Health Division of Country Health Policies and Systems World Health Organization Regional Office for Europe, Copenhagen, Denmark; 3 Sciences Po, School of Public Affairs, Paris, France; 4 Department of Health Sciences and Technology, Chair of Bioethics, ETH Zurich, Zurich, Switzerland

**Keywords:** Artificial intelligence, Health, Tools, Governance, AI ethics, Practical approaches

## Abstract

The rapid advancement of artificial intelligence (AI) sparked the development of principles and guidelines for ethical AI by a broad set of actors. Given the high-level nature of these principles, stakeholders seek practical guidance for their implementation in the development, deployment and use of AI, fueling the growth of practical approaches for ethical AI. This paper reviews, synthesizes and assesses current practical approaches for AI in health, examining their scope and potential to aid organizations in adopting ethical standards. We performed a scoping review of existing reviews in accordance with the PRISMA extension for scoping reviews (PRISMA-ScR), systematically searching databases and the web between February and May 2023. A total of 4284 documents were identified, of which 17 were included in the final analysis. Content analysis was performed on the final sample. We identified a highly heterogeneous ecosystem of approaches and a diverse use of terminology, a higher prevalence of approaches for certain stages of the AI lifecycle, reflecting the dominance of specific stakeholder groups in their development, and several barriers to the adoption of approaches. These findings underscore the necessity of a nuanced understanding of the implementation context for these approaches and that no one-size-fits-all approach exists for ethical AI. While common terminology is needed, this should not come at the cost of pluralism in available approaches. As governments signal interest in and develop practical approaches, significant effort remains to guarantee their validity, reliability, and efficacy as tools for governance across the AI lifecycle.

**Supplementary Information:**

The online version contains supplementary material available at 10.1007/s43681-024-00563-x.

## Introduction

The rapid advancements in artificial intelligence (AI) and the ethical challenges involved have resulted in a proliferation of guidelines to aid the development and deployment of ethical, trustworthy and responsible AI. These guidelines, produced by a range of actors such as national governments, private companies, and international organizations, set out broad high-level principles, but have so far paid limited attention to how these principles are to be applied or enforced [1]. Further, while representing a crucial first step on which the development of laws, regulation and standards of AI can build on [2, 3], studies have shown that AI ethics guidance suffers low rates of adoption in practice [3–5]. Ethical, principle-based guidance is commonly described as vague, too general and high-level [2, 6, 7], and as largely lacking mechanisms to facilitate enforcement or translation into practice [1, 8]. In an analysis by AlgorithmWatch of more than 160 documents, only ten include practical enforcement mechanisms [8]. This has prompted a call for a transition “from what to how” in AI ethics [9].

Over the last years, substantial work has thus been dedicated to “lowering the level of abstraction” [10] and to translating ethical principles into actionable and specific practical requirements in AI governance [6, 11]. This has spurred the development of a myriad of practical approaches aiming at providing guidance on ethical AI. Among some of the early, most prominent examples, the Assessment List for Trustworthy AI (ALTAI) is a practical tool developed by the High-Level Expert Group on Artificial Intelligence (AI HLEG), appointed by the European Commission, to translate their Ethics Guidelines for Trustworthy Artificial Intelligence into a self-assessment checklist for developers and deployers [12]. More recently, the United Nations Educational, Scientific and Cultural Organization (UNESCO) developed their own ethical impact assessment tool [13] to ensure that the development of AI aligns with their Recommendation on the Ethics of AI [14]. Additionally, several governments and public institutions have made extensive efforts to develop frameworks or tools aiming to aid the assessment of the possible impacts of the use of AI (see e.g., the Finnish “Assessment framework for non-discriminatory AI systems” [15], the Ada Lovelace Institute Algorithmic Impact Assessment for AI in healthcare [16], and the Dutch “Fundamental Rights and Algorithms Impact Assessment (FRAIA)” [17]). A number of private companies have also developed open-source tools for the assessment and improvement of the trustworthiness of AI systems (see e.g. Holistic AI Open Source Library [18]) or conformity with standards (e.g. Saimple [19]).

The high number and variety of practical guidance tools and approaches being developed by public and private organizations are reflected in the continuously growing catalog of tools and metrics for trustworthy AI by the Organisation of Economic Co-operation and Development (the OECD Catalogue) which, at time of writing, listed 703 technical, educational and procedural tools (stand: March 2024) designed to aid AI actors in the development and deployment of trustworthy AI systems and applications [20]. The OECD Catalogue represents a highly heterogeneous collection of frameworks, codes, toolkits, checklists, software, standards, guidelines, agreements, developed by a broad set of stakeholders, varying by target group, users, sectors, and purpose.

Along with rules, processes, and procedures, such approaches and tools can aid ensuring legal compliance in the development, deployment and use of AI, as well as adherence to social and ethical standards [21]. The original proposed AI Act [22] legislation (21.4.2021), for example, refers specifically to “harmonized standards and supporting guidance and compliance tools” as enablers of compliance in the development, deployment and use of ethically sound AI. These developments signal the growing need for practical guidance. At the same time, the highly heterogeneous landscape and the patchwork of approaches and tools sound warning bells as there appears to be a lack of consensus about what these tools should achieve, how they are validated, and how they should operate.

Focusing on AI for health, we therefore set out to examine and synthesize evidence from literature reviews of the types of practical approaches available, understand how and by whom they have been developed, for what purpose, whether they have been validated and against what criteria, their limitations, gaps, and whether their impact is known. We use the term “practical approach” to capture all tools, toolkits, frameworks, guidance, and methods available for the promotion of ethical, trustworthy and responsible AI in practice. Our research was further guided by the following considerations: first, if any of these practical approaches are to be widely adopted, their promise should be substantiated by their effectiveness; second, if they are developed as a means to assist organizations in complying with ethical standards, information on their provenance and vetting should be easily accessible and verifiable; third, if use of any such approach aims to give users, or more broadly consumers, assurance of an ethical AI product, it should be clear on what basis this assurance is possible.

Given the heterogeneity in the literature, we opted for a scoping review of existing review/survey articles. This type of review is considered helpful for mapping and assessing the breadth and focus of a body of literature on a particular subject [23], to identify gaps and specific research questions in emerging fields [24, 25], and also to gather important insights into the ways, concepts or terms have been used [26]. In the case of complex and diverse literature, scoping reviews are particularly useful [27].

## Methodology

We performed a scoping review of peer-reviewed scholarly and gray literature on the practical approaches available for the promotion of ethical, responsible, and trustworthy AI in health published between 2019 and 2023. This scoping review follows the PRISMA extension for scoping reviews (PRISMA-ScR) [28]. The data collection process consists of four steps: identification, evidence screening, eligibility and data capture.

### Search and identification

We examine peer-reviewed and gray literature review/survey articles (literature produced without peer-review by government, academics, business and industry in electronic and print formats). Records were searched for between February and May 2023 in the languages English, German, French, Spanish, Italian, Norwegian, and Finnish.

We adopted a multi-step, systematic and comprehensive search strategy that covered both multidisciplinary and more specific databases and search engines of peer-reviewed and non-peer reviewed literature such as pre-prints and conference articles. As a first step, the initial search was conducted in three databases: Scopus (covering among others the database MEDLINE), Web of Science, and Google Scholar. In a second step, we conducted searches of databases serving specific fields such as IEEExplore (engineering and technology), MedRxiv (medicine), and arXiv (natural sciences, engineering, and economics). The search strategy can be found in the Supplementary Files.

**Fig. 1:**
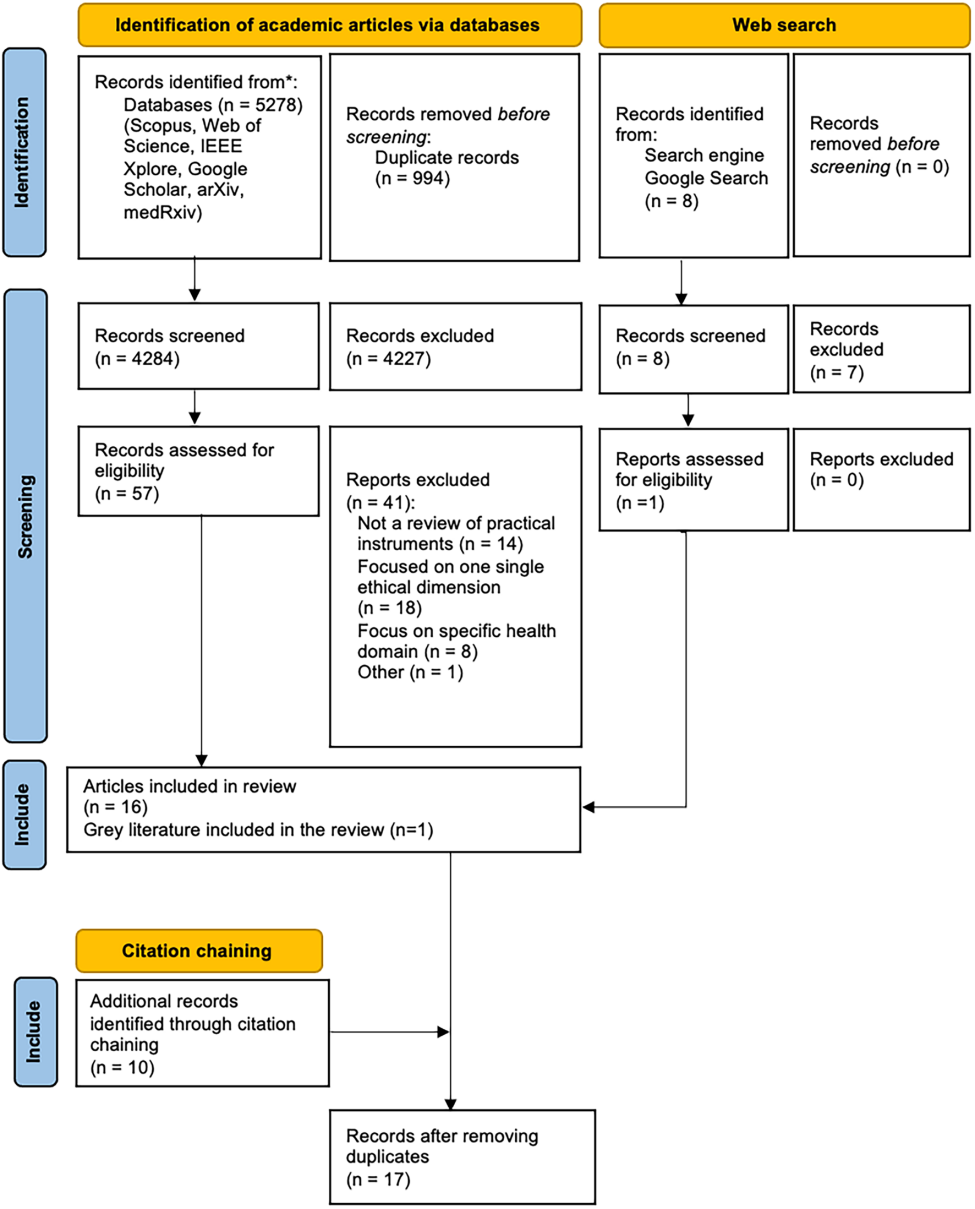
Adapted PRISMA-ScR (Preferred reporting items for systematic reviews and meta-analyses) 2020 flow diagram for new scoping reviews [29]

Search strings include terms related to reviews (e.g. also survey) of tools or frameworks (incl. standards, checklists, toolkits, assessment, audits, impacts or practical approaches) for ethical (incl. responsible and trustworthy) AI in health domains (e.g. healthcare, medicine, mHealth, digital health). The third step consisted of a Google web search. This search was performed using various terms related to the review of practical approaches for ethical, trustworthy, or responsible AI in health and was conducted using private browsing mode, after logging out from personal accounts and erasing all web cookies and history. For each search, the 100 first search results were followed and screened for relevance. This added one further non-duplicate record to the body of documents. Finally, we exhausted the practice of citation chaining and examined the reference lists of all of the selected documents and identified one additional non-duplicate document. In total 4284 records (5278 before removing duplicates with Rayyan (web version, Rayyan Systems Inc., Cambridge, MA)) were retrieved. A log of the search strategies and results were kept in a Word-document (Microsoft Word for Mac, version 16.74, Microsoft, Seattle, WA). This process is displayed in Fig. 1. With this broad search strategy, we aimed to reduce the risk of missing relevant documents.

### Screening

The articles identified with the help of academic databases were screened for relevance based on title and abstract by SK, EV, and ER. The documents retrieved through web search were screened following a two-step process. SK first screened their title and summary, and second, retained the documents that reviewed tools aimed at promoting ethical/responsible/trustworthy AI, which were not academic articles. Finally, SK identified documents through citation chaining based on document titles and abstracts. All documents not reviewing, surveying or assessing practical approaches to advance ethical AI were excluded, leaving a body of 57 documents for which we retrieved the full-text documents.

### Eligibility and selection

The documents were independently assessed for eligibility by the three authors. Aiming to examine the landscape of practical approaches to AI ethics in health, our inclusion and exclusion criteria were the following. Documents were included that (1) reviewed more than one practical approach, and (2) focused on ethical/responsible/trustworthy AI in general or in health. The first eligibility criteria specifies that the articles should not merely examine a single approach. Further, we do not only include reviews that narrowly focus on practical approaches for AI in health but also on those that focus on approaches for AI in general, as they may be relevant for AI in health. We excluded records reviewing or evaluating approaches focused only on one specific ethical principle (for example fairness) as furthering ethical, responsible or trustworthy AI requires consideration of more than one ethical principle. Although we do not discount the value of these individual approaches, we aimed at understanding the landscape of comprehensive proposals. Finally, records that were published in another language or which were not an article, but a book, full thesis, magazine, or newspaper article, were also excluded.

Any disagreements on selection were resolved through discussion and consensus among the authors. This assessment of eligibility resulted in a body of 19 documents. The full-text articles were then further reviewed jointly by the three researchers, leading to the inclusion of a total of 17 documents in the final analysis.

### Analysis and data capture

The articles were analyzed by all three researchers to identify common topics or descriptors. The analysis identified two main clusters in the corpus of the articles: one on the characteristics of the practical approaches, and one on barriers to adoption. In a second step, following a deductive approach, we analyzed the documents guided by the following four questions: Which types of practical approaches are available for ethical AI? By whom have they been developed, and for what purpose? Where along the AI lifecycle are they meant to be used and by whom? What are the main barriers to their adoption? The analysis consisted of an iterative process performed using Nvivo (1.7.1 (4844), Lumivero, Denver).

## Results

The final body of documents includes 17 articles, published between 2020 and 2023, with a majority between 2022 and 2023 (see Table 1). Of all of the articles, 15 were published in scientific journals, one was a working paper, and one was an extract of gray literature. Nine of the articles focus on tools for AI in general [9, 30–37] and eight on AI in health [38–45]. The number of practical approaches examined by the articles vary significantly, ranging from 6 to 121.

**Table 1:** Final sample of articles analyzed

Key	Authors	Focus on health	Types of practical approaches examined	
[33]	Ayling, J. and Chapman, A. (2022)	No	Audit and assessment tools	
[35]	Boza, P. and Evgeniou, T. (2021)	No	Software toolkits and frameworks documentation processes and tools for auditing	
[32]	Crockett, K. A. et al. (2021)	No	Toolkits for practical application in SMEs	
[43]	Crossnohere, N. L. et al. (2022)	Yes	Frameworks and checklists offering guidance on applying or evaluating AI in medicine	
[42]	De Hond, A. A. H. et al. (2022)	Yes	Actionable guidance for AI-based prediction models development, evaluation and implementation	
[38]	Garbin, C. and Marques, O. (2022)	Yes	Methods and tools to promote auditing and transparency of datasets and models in ML for healthcare applications	
[39]	Goirand, M., Austin, E. and Clay-Williams, R. (2021)	Yes	Technical checklists, organizational and/or evidence-based approaches	
[31]	Kaur, D. et al. (2022)	No	Methods, techniques, toolkits, and guidelines	
[41]	Lehoux, P. et al. (2023)	Yes	Practice-oriented tools, defined as frameworks and/or sets of principles with clear operational explanations	
[45]	Marwood, T. et al. (2022)	Yes	Frameworks and a toolkit for the application of AI in healthcare in Australia	
[36]	Minkkinen, M., Laine, J., and Mäntymäki, M. (2022)	No	AI Auditing tools and frameworks	
[9]	Morley et al. (2020)	No	Tools and methods to help developers, engineers and designers of ML	
[44]	Pradhan, K.B. and Sandhu, N. (2020)	Yes	Frameworks for responsible AI innovation in healthcare	
[30]	Prem, E. (2023)	No	Methods and tools (generally defined as Approaches)	
[40]	Solanki, P., Grundy, J., and Hussain, W. (2023)	Yes	Guidelines, frameworks, and technical solutions for operationalising ethics in AI for healthcare	
[34]	Tidjon, L. and Khomh, F. (2023)	No	Practical guidance of ethical AI principles	
[37]	Wong, R.Y., Madaio, M. A, and Merrill, N. (2023)	No	Ethical Toolkits (understood as curated collections of tools and materials)	

In the following sections, we aim to answer the research questions and examine the main characteristics of the practical approaches reviewed and the barriers to their adoption.

### Characteristics of practical approaches for ethical AI

Most articles in our sample set out to examine the practical approaches available, their coverage, their intended users, and the gaps in the current landscape. In total, 15 of the articles classify or organize the reviewed approaches along different dimensions. As an illustration, Ayling and Chapman [33] use the typologies developed from their literature review of sectors, stakeholders, historical practice, and stages in the AI production process as a basis for their classification, while Prem [30] reviews them according to the ethical principles addressed, approach category, practicability, and point of intervention in the AI lifecycle.

### Types of practical approaches available for ethical AI

We identified a sizeable and highly heterogeneous body of different practical approaches to help guide ethical implementation. These include not only ‘tools, checklists, procedures, methods, and techniques’ but also a range of far more general approaches that require interpretation and adaptation such as for research and ethical training/education as well as for designing ex-post auditing and assessment processes. Together, this body of approaches reflects the varying perspectives on what is needed to implement ethics in the different steps across the whole AI system lifecycle from development to deployment. The more than 46 terms used to capture these various practical approaches are rarely defined, and their use is diverse across the examined literature.

The usage of certain terms, e.g. tools and frameworks, is inconsistent across the literature. They are both used as umbrella terms more generally as well as to describe specific approaches. In some of the papers, “tools” is used as a technical term that may specify technical, documentation, implementation or audit and impact assessment tools [33–35], while elsewhere it is used as an umbrella term that also includes toolkits [33, 41]. Similarly, the term “framework” is used to specify both practical tools for application [33] and conceptual models [30, 36]. Occasionally the term is used interchangeably with “guideline” (see e.g. [43],). This ambiguity also applies to the distinction between “tools” and “toolkits”. “Toolkits” are by some considered to be collections of resources [32] that include “tools” [37], by others it is understood as a type of “tool” [9, 35, 36].

### Provenance and purpose of practical approaches

Information on the provenance of the practical approaches is reported only by three out of the 17 articles examined [33, 37, 41]. These articles cite technology companies, university centers and academic researchers, non-profit organizations or institutes, open-source communities, design agencies, and government agencies. The private sector has been particularly active in developing the available practical approaches closely followed by academia [33, 37, 41]. A small share of approaches are developed through multisectoral efforts [41].

The diversity of stakeholders developing practical approaches is reflected in the wide range of their intended purposes. While the main objective is to ensure that AI systems are developed, deployed and used in alignment with ethical principles, one study identifies up to 40 distinct purposes. These range from guiding implementation of prominent ethical principles such as nonmaleficence, transparency, privacy and beneficence, to assisting in putting in place ethical data management and addressing feasibility, acceptability and interoperability [41]. Most approaches reviewed by the articles aim to equip stakeholders with the necessary tools or knowledge to address one or few ethical principles [30]. Practical approaches most often seek to advance fairness/bias [9, 30–32, 34, 41], transparency [32, 39, 41, 43, 45], privacy [30, 32, 39, 41], explainability [9, 30, 31], and accountability [30–32].

### Availability of practical approaches throughout the AI lifecycle and target users

The majority of the approaches examined are intentionally designed to aid AI actors at specific stages in the development, deployment and use of AI systems and applications. There is a higher prevalence of practical approaches to guide the design [30, 33, 39] and development of AI systems [38, 43]. Few tools have been developed for the later stages of the AI lifecycle such as monitoring [30, 32, 38, 43], and audit and compliance [32, 41] of AI systems. Whereas frameworks are common for implementation in the design phase, audits, checklists and metrics are more common in the test phase [30]. Finally, it appears that these practical approaches address different ethical principles at various stages of the lifecycle [see e.g., [9, 31, 43]]. To advance explainability, accountability or fairness, for example, most approaches for explainability target the early modeling phases, approaches for fairness are meant to be implemented in the data collection and deployment phase, while approaches promoting accountability often target the planning stage [31].

With regard to target users, most frequently practical approaches target actors involved in the development of AI systems, their delivery, and in quality assurance [33]. Certain categories of practical approaches target distinct groups. Toolkits, for example, are largely aimed at developers, data scientists, designers, technologists, implementation or product teams, analysts and UX teams [37]. Intended users of impact assessment and auditing approaches are mainly decision-makers or actors involved in oversight [33]. Only a few practical approaches target multiple hierarchical levels within organizations [37] or stakeholders outside companies involved in policymaking, governments, civil society organizations, community groups, or users [32, 37]. Few approaches include the broader public in the application of their toolkits [32]. Even where the inclusion of a broad set of stakeholders is a stated aim, guidance is lacking on how to do so in practice [37, 43].

### Barriers to adoption

The articles reviewed here highlight four primary impediments to the adoption and implementation of practical approaches (see Table 2):

1. Skills: Practical approaches are often difficult to use, which can discourage adoption.
2. Absence of Guidance: There is often a lack of clear instructions or support for implementing practical approaches. Without proper guidance, users may struggle to understand how to apply these methods in their specific contexts.
3. Lack of Evaluation Mechanisms and Metrics: Without robust evaluation mechanisms and metrics, it is challenging to assess the effectiveness and impact of practical approaches.
4. Limited Awareness: There is often insufficient awareness of practical approaches among potential users. This can stem from inadequate dissemination of information or a lack of exposure to these methods to relevant target userls.

**Table 2:** Obstacles to the implementation of ethical AI practical approaches

High level of skills, resources and effort required for use	[9, 30, 32, 33, 37]
Absence of documentation, specific instructions, examples, case studies or training materials or courses	[32, 34]
Lack of evaluation mechanisms and metrics for success	[31, 39]
Limited awareness of practical approaches	[40]

Limited usability is the most widely cited impediment to the adoption of such approaches. Many practical approaches require a relatively high level of skills, resources and effort to be adopted [9, 30, 32, 33, 37]. The absence of sufficient guidance on how to implement them further impairs their usability [9, 30, 32].

The highly technical nature of toolkits, for example, often necessitates technical skills for their effective utilization. One study finds that, in practice, the actual design and functionality of the majority of toolkits are focused on technical approaches in enacting ethics in AI [37]. This makes it challenging for users without a technical background, e.g. project managers, lawyers or other non-technical stakeholders, to employ such toolkits, or at least presupposes a sufficient level of technical knowledge [37]. Specifically examining small- and medium-sized businesses, another study indicates that the application of toolkits is resource-intensive, demanding substantial time investments from staff resulting in additional workloads [32]. The financial and non-financial costs that may arise for small-sized businesses or organizations in implementing these toolkits can, in practice, act as a disincentive and limit their adoption [9]. Additionally, a large proportion of approaches are general in nature, often aiming at clarifying ethical principles or guiding practitioners with very broad suggestions, lacking specific practical guidance [30].

Many toolkits do not include case studies, use cases, or training material that could facilitate practical application [32]. The most common potential target users such as developers and data scientists, without further guidance and background knowledge in the ethics of AI, would struggle to implement these approaches effectively. Even with standardized approaches and processes [33], applications such as for the assessment of AI models, still would require users to have an understanding of and ability to effectively use the outputs.

More specifically, one study indicates that more than 80 percent of toolkits do not provide educational resources on how to apply them in an organization [32]. Even where courses on AI ethics are made available, another study argues that they most commonly focus on guidelines and standards, rather than raising awareness about the available practical approaches for their effective implementation [34]. This gap in practical guidance, as noted, is particularly challenging for small- and medium-sized businesses, which may require comprehensive training and support materials to successfully implement ethical AI toolkits due to their limited resources [32].

The lack of standards, evaluation mechanisms and measures of successful implementation represents a further obstacle to the adoption of practical approaches [31, 39]. Defining clear indicators for success is essential to assess the efficacy of approaches and determine if they are fit for purpose [39]. Most approaches do not report on whether a formal methodology was used for their development, and none cast light on their real-world applicability and usability and on how their validity, reliability and relevance was ensured [41]. According to one author, less than one-third of practical approaches directly address how to evaluate their successful implementation [39].

While users want concrete, measurable evidence that AI systems meet certain ethical criteria, there is a lack of agreed indicators or systems to test or evaluate these approaches in a way that potential users can understand and trust [31]. Additionally, a lack of awareness also hinders the implementation of such approaches [40]. This lack of awareness may stem from the novelty of practical approaches [31, 39], which may also hinder their adoption.

## Discussion and conclusion

Our scoping review uncovers a heterogeneous and intricate ecosystem of practical approaches, characterized by diverse and inconsistent terminology as well as lack of consensus on their defining features such as purpose and target audience. At the moment, there appears to be no common understanding of what “tools”, “toolkits” and “frameworks” for ethical AI entail. Clearly defined categories of approaches, and an enhanced understanding of their purpose and capacity, are crucial for policymakers if these approaches are to be implemented for the governance of AI. At the same time, the diversity in terminology, practical approaches, and ethical principles covered by them, implies that there is no single approach to promote AI ethics. The implementation of approaches requires a thorough understanding of the context within which the AI will operate and the potential ethical concerns that must be addressed.

While there is a need to streamline terminology and the understanding of what each specific approach entails, this convergence should not happen at the cost of plurality as some approaches may be more suitable than others depending on context and purpose. There is indeed considerable variation in the way the various practical approaches apply across the AI lifecycle. Few, however, cover all or multiple stages of the AI lifecycle. A substantial share is developed for practical guidance to the earlier phases of the AI lifecycle, i.e., the design and development phases. Practical approaches to guide use and monitoring are largely absent. This may reflect the private sector’s prominent role in both designing and developing AI systems as well as in the creation of practical approaches. Private companies have strong incentives to assess the adequacy of their governance mechanisms in the absence of clear norms or rules and to prevent reputational-related risks. Considering the imperative of aligning AI systems with ethical standards over the entire lifecycle, the accumulation of practical approaches at the early stages highlights the need for a lifecycle-perspective, given the interconnectedness of the different phases. A lifecycle perspective ensures that the potential ethical risks and trade-offs and/or unintended consequences are addressed across the whole process. The existing landscape of practical approaches provides, however, limited assistance for this task.

These considerations bring up three questions:

First, whether the development of practical approaches to AI ethics is emerging as a business opportunity. While this could foster the production of a wide range of available approaches, without proper evaluation and testing of their performance, they are unlikely to sufficiently guide the translation of ethics into practice. The dominance of a few stakeholders such as the private sector and academia in their development has resulted in the “narrowing” down of the ethical requirements addressed by such approaches [46], not reflecting the full breadth of ethical principles. For example, some authors have documented a disproportionate proliferation of approaches addressing specific ethical concerns such as privacy, explainability, fairness [9, 30, 46] and accountability [46].

Second, whether these practical approaches are robust and rigorous enough to be used for monitoring of AI systems and their oversight. While auditing and impact assessment have attracted much attention from researchers, companies, and policymakers, and are considered critical for understanding and minimizing harms from AI systems, there is a paucity of practical approaches for these purposes. This may be symptomatic of the current level of maturity of standards for these processes, largely because of the evolving regulatory environment [47]. Due to the novelty of such practices, professional norms facilitating their implementation are still largely absent [48].

Third, whether effective governance of AI may necessitate context-specific, tailored approaches to ensure adequate oversight. A recently suggested approach involves the development of standards for “ethical disclosure by default” [49]. Rather than imposing uniform ethical norms, this approach would require AI system providers to adhere to minimum standards for procedural consistency in technical testing, documentation, and public reporting. This approach would ensure AI systems are transparent and accountable by design and that users and stakeholders are fully informed about the system's operations, risks, and impacts. This would shift ethical decision-making to stakeholders while limiting the discretion of providers in addressing complex ethical normative issues in the development of AI products and services [49].

Apart from the observed patterns in the current landscape of approaches, the review highlights the presence of significant barriers to their adoption, corresponding to reports of their limited implementation [32, 39, 45]. These include high levels of skills, knowledge and resources required for adoption, the lack of awareness of practical approaches, and the absence of methods or approaches to the measurement of their successful implementation. Measuring successful implementation, as also noted by others [39], is crucial for the assessment of the effectiveness and efficiency of these approaches in advancing ethical AI. There is a clear need for relevant, and practical validation metrics based on standards to assess AI systems’ compliance with ethical principles [50]. However, measuring the impact and success of AI ethics in practice remains challenging [9, 51].

Considering that practical approaches are developed largely in the absence of a formal methodology, our understanding of whether they do what they intend and claim to do is limited. Given the diversity of practical approaches and their purposes, the appropriate validation criteria are also likely to vary. Suitable criteria may address their effectiveness and impact, reliability, usability, and stakeholder acceptance.

For the first criteria, the effectiveness and impact of practical approaches on ethical outcomes, quantitative metrics and ethical benchmarks can be useful. Several metrics have recently been proposed, e.g. for fairness [52], which could be used to assess and ensure that such approaches effectively promote ethical principles. Such metrics may also be useful for assessing the reliability and consistency of practical approaches across different contexts. Given the existing barriers to adoption, usability and stakeholder acceptance should be considered as further critical criteria in the validation of approaches. Relevant metrics could include the ease of implementation or the quality of documentation that aids adoption, positive stakeholder feedback or confidence in the practical approach.

While the validation of practical approaches and the measurement of their successful implementation is essential, the low level of their adoption also raises questions concerning incentives: Which incentives are needed to encourage adoption? Will only practical approaches that have “teeth” and aid legal compliance be implemented? Governments are currently taking first steps to enshrine the ethics of AI and data into law. The Danish government adopted a law on disclosure of data ethics, requiring Denmark’s largest companies to provide information on compliance with their data ethics policy as part of their annual reporting. Following this, together with business and consumer organizations, the government created a labeling program for IT security and responsible use of data [53]. In Canada, the government has made risk assessment a mandatory step in the design and deployment of systems for automated decision-making. For this purpose, the Algorithmic Impact Assessment Tool [54] was developed. While governments increasingly show interest in and develop these types of practical approaches, much effort still is required to ensure their validity, reliability, and effectiveness if they should be conceived of as governance tools.

## Supplementary Information

Below is the link to the electronic supplementary material.

Supplementary file1 (DOCX 25 KB)

Supplementary file2 (DOCX 18 KB)
