# Supplementary material for: The rise of checkbox AI ethics: a review
Source: AI Ethics. 2024 Sep 5;5(3):1931–40. doi: 10.1007/s43681-024-00563-x (PMC12103313; doi:10.1007/s43681-024-00563-x)
Supplement: Supplementary file 1 — Supplementary file1 (DOCX 25 KB) [file 43681_2024_563_MOESM1_ESM.docx]

Content analysis

| Table S2: Coded documents (n=17) |
| --- |
|  |

|  | | | |  | | | |  | | | |  |  |  |  |  |  |  |  |  |
| --- | --- | --- | --- | --- | --- | --- | --- | --- | --- | --- | --- | --- | --- | --- | --- | --- | --- | --- | --- | --- |
|  | **Characteristics** |  | **Total** | [32] | [34] | [31] | [42] | [41] | [37] | [38] | [30] | [40] | [44] | [35] | [9] | [43] | [29] | [39] | [33] | [36] |
|  |  | Ethical principles | 9 | ○ | ○ | ● | ● | ○ | ○ | ● | ● | ● | ● | ○ | ● | ○ | ● | ○ | ● | ○ |
|  |  | Stages | 11 | ● | ○ | ● | ● | ● | ● | ● | ● | ● | ○ | ○ | ● | ○ | ● | ● | ○ | ○ |
|  |  | Creator | 3 | ● | ○ | ○ | ○ | ○ | ○ | ○ | ○ | ● | ○ | ○ | ○ | ○ | ○ | ○ | ○ | ● |
|  |  | Target user | 3 | ● | ○ | ● | ○ | ○ | ○ | ○ | ○ | ○ | ○ | ○ | ○ | ○ | ○ | ○ | ○ | ● |
|  | **Barriers to adoption** | Skills, resources and effort required | 5 | ● | ○ | ● | ○ | ○ | ○ | ○ | ○ | ○ | ○ | ○ | ● | ○ | ● | ○ | ○ | ● |
|  |  | Absence of instructions or training | 2 | ○ | ○ | ● | ○ | ○ | ○ | ○ | ○ | ○ | ○ | ○ | ○ | ○ | ○ | ○ | ● | ○ |
|  |  | Lack of standards, evaluation mechanisms and metrics for success | 3 | ○ | ○ | ○ | ○ | ○ | ○ | ● | ● | ○ | ○ | ○ | ○ | ○ | ○ | ○ | ○ | ○ |
|  |  | Limited awareness of practical approaches | 1 | ○ | ○ | ○ | ○ | ○ | ○ | ○ | ○ | ○ | ○ | ○ | ○ | ○ | ○ | ● | ○ | ○ |
