# Supplementary material for: The rise of checkbox AI ethics: a review
Source: AI Ethics. 2024 Sep 5;5(3):1931–40. doi: 10.1007/s43681-024-00563-x (PMC12103313; doi:10.1007/s43681-024-00563-x)
Supplement: Supplementary file 2 — Supplementary file2 (DOCX 18 KB) [file 43681_2024_563_MOESM2_ESM.docx]

**Example full search string**

| **Scopus** (final search date: 23.2.2023, 2204 documents identified) |
| --- |
| ( TITLE-ABS-KEY ( ( review  OR  survey )  AND  ( tool*  OR  framework*  OR  guid*  OR  standard  OR  checklist*  OR  toolkit*  OR  assess*  OR  audit  OR  impact  OR  "by design"  OR  "practical approach"  OR  "in practice" ) ) )  AND  ( TITLE-ABS-KEY ( ethical  OR  ethics  OR  principles  OR  trust*  OR  fair*  OR  justice*  OR  responsible  OR  equit*  OR  transparen*  OR  account*  OR  "non-maleficence"  OR  safe  OR  secur*  OR  "data ethics" ) )  AND  ( TITLE-ABS-KEY ( "artificial intelligence"  OR  "AI"  OR  "machine learning" ) )  AND  ( TITLE-ABS KEY ( health  OR  medic*  OR  healthcare  OR  mhealth  OR  "digital health"  OR  pharma* ) )  AND  ( EXCLUDE ( DOCTYPE ,  "sh" )  OR  EXCLUDE ( DOCTYPE ,  "no" )  OR  EXCLUDE ( DOCTYPE ,  "ed" )  OR  EXCLUDE ( DOCTYPE ,  "er" )  OR  EXCLUDE ( DOCTYPE ,  "bk" )  OR  EXCLUDE ( DOCTYPE ,  "le" )  OR  EXCLUDE ( DOCTYPE ,  "tb" ) )  AND  ( LIMIT-TO ( PUBYEAR ,  2023 )  OR  LIMIT-TO ( PUBYEAR ,  2022 )  OR  LIMIT-TO ( PUBYEAR ,  2021 )  OR  LIMIT-TO ( PUBYEAR ,  2020 )  OR  LIMIT-TO ( PUBYEAR ,  2019 ) )  AND  ( LIMIT-TO ( LANGUAGE ,  "English" )  OR  LIMIT-TO ( LANGUAGE ,  "German" )  OR  LIMIT-TO ( LANGUAGE ,  "Italian" )  OR  LIMIT-TO ( LANGUAGE ,  "Spanish" )  OR  LIMIT-TO ( LANGUAGE ,  "Norwegian" )  OR  LIMIT-TO ( LANGUAGE ,  "Danish" )  OR  LIMIT-TO ( LANGUAGE ,  "Greek" )  OR  LIMIT-TO ( LANGUAGE ,  "Finnish" )  OR  EXCLUDE ( LANGUAGE ,  "Chinese" )  OR  EXCLUDE ( LANGUAGE ,  "Russian" ) ) Refined to document type: Review, article, conference paper, conference review, book chapter |
